# Dexamethasone implant confined in Berger’s space

**DOI:** 10.1186/s40064-016-3490-9

**Published:** 2016-10-13

**Authors:** Pierre Dubrulle, Franck Fajnkuchen, Lise Qu, Audrey Giocanti-Aurégan

**Affiliations:** 1 Ophthalmology Department, DHU Vision and Handicaps, Avicenne Hospital, 125 Rue de Stalingrad, 93000 Bobigny, France; 2 Centre d’imagerie et de Laser, 11 Rue Antoine Bourdelle, 75015 Paris, France

**Keywords:** Diabetic macular edema, Ozurdex implant, Treatment complications, Cataract

## Abstract

We reported a new complication after dexamethasone implant injection. The patient was treated by dexamethasone implant injection for diabetic macular edema. After injection, we report an implant stucked in Berger’s space behind the crystalline lens capsule in the visual axis. This situation resolved after cataract surgery and the implant moved to a less inconvenient localization.

## Background

Dexamethasone implant is currently largely used in the treatment of diabetic macular edema, macular edema secondary to vein occlusions, uveitis or Irvine Gass syndrome. Efficacy and safety of this treatment are well documented. Complications of the implant injections such as migration in the anterior chamber throught iridotomy or in case of aphakia have already been described (Stepanov et al. 2015). Accidental injections into the crystalline lens (Coca-Robinot et al. 2014; Fasce et al. 2014) have also been reported. We describe here another complication after injection: the confinement of the implant in Berger’s space in the visual axis.

## Case presentation

We report here the case of a 63 year-old diabetic woman presenting for diabetic macular edema and cataract. There was no history of previous blunt trauma to the eye. She was on oral hypoglycemic medications for her diabetes. On ophthalmological examination, best-corrected visual acuity was 20/63 in both eyes. The slit lamp examination revealed a bilateral cataract. Intraocular pressure (IOP), measured by Goldmann applanation tonometry was 19 mm Hg in each eye. Discs were healthy and not cupped. There was a severe no proliferative diabetic retinopathy in both eyes. We diagnosed a central diabetic macular edema on her left eye and decided to treat the edema before operating on cataract. A Dexamethasone implant injection was performed on her left eye. The injection technique and execution were unremarkable. The needle was inserted 4 mm away from the limbus in the inferior and temporal quadrant, directly, without scleral path towards the center of the eye. The needle was advanced until the sleeve touches the conjunctiva, and then the implant was injected.

**Fig. 1:**
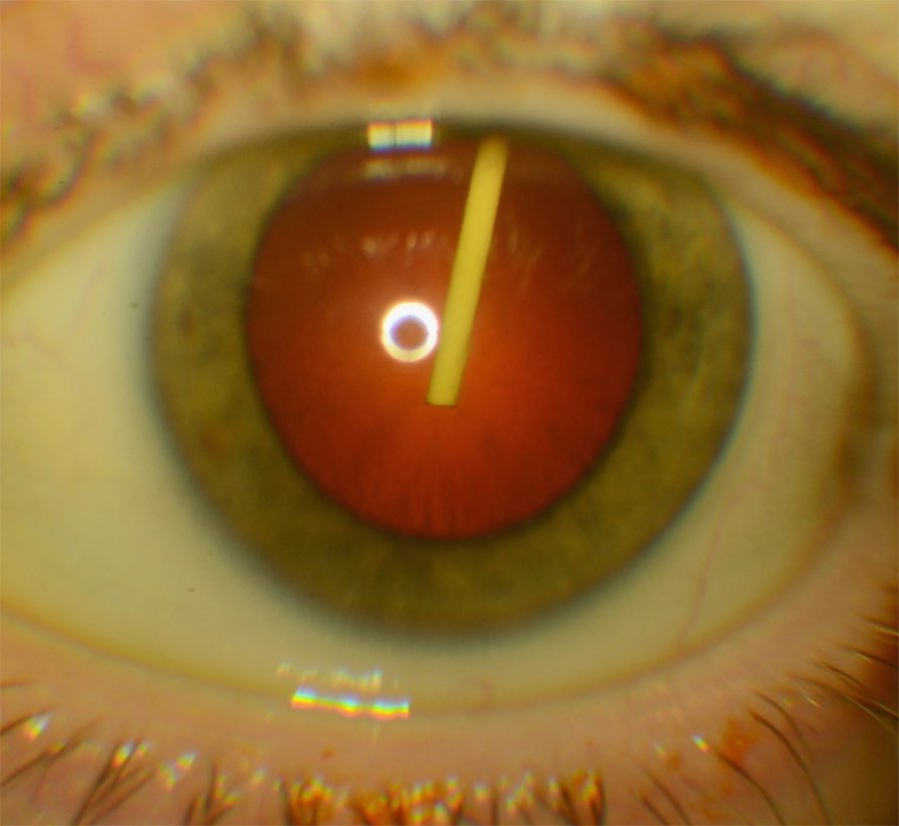
Slit lamp photography of a 63 year old diabetic woman with dexamethasone implant confined in Berger’s space, between the anterior hyaloid and posterior lens capsule

Two days after injection, the patient was referred to the department complaining for floaters. On ophthalmological examination, best-corrected visual acuity was 20/63 in the right eye and decreased at 20/125 in the left eye. The slit lamp examination after dilatation revealed the dexamethasone implant confined just behind the posterior capsule (Fig. 1). The implant only showed a little mobility and did not exit of this confined localization. Whatever the position of the patient, prone position or seated, the implant was always visible in the pupillary area without slit lamp, and did not change position. Examination of the fundus was unremarkable except for the severe non proliferative diabetic retinopathy. IOP measured by Goldmann was 21 mmHg in the left eye and a preventive combination of timoptol and dorzolamide was prescribed twice a day. The scheduled cataract surgery of the left eye was performed one month after Dexamethasone injection and was unremarkable. There was no acceleration of cataract development, and no evidence of lens traumatism visible during surgery.

**Fig. 2:**
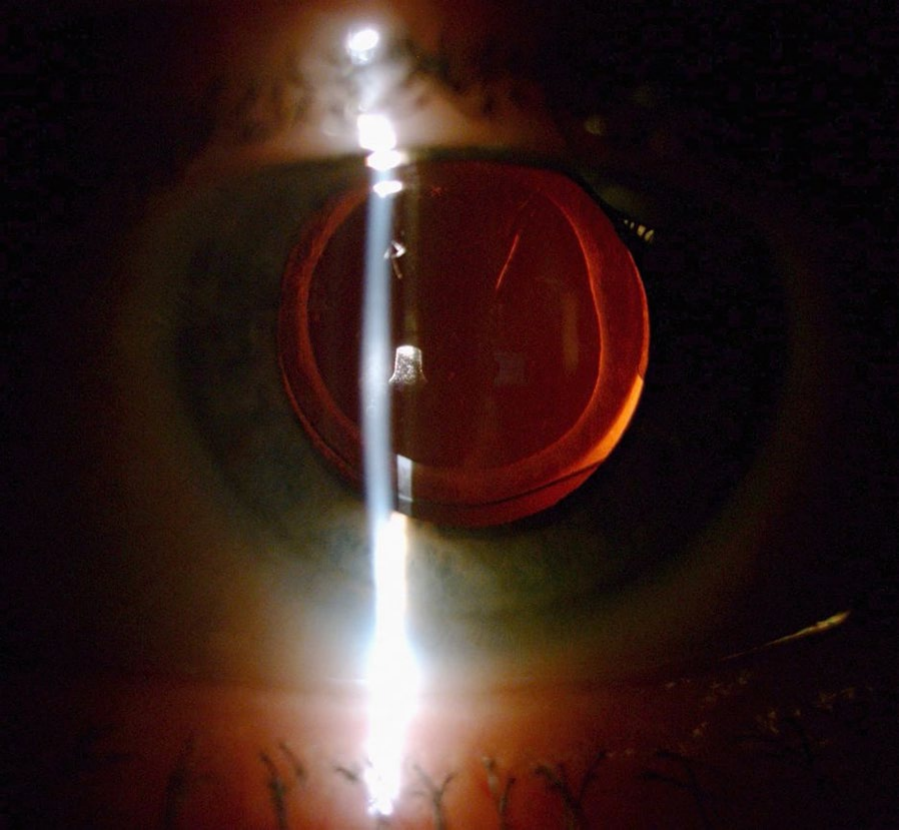
Slit lamp photography, after cataract surgery. Dexamethasone implant is not visible in Berger’s space

Seven days after surgery, in the left eye, best-corrected visual acuity was 20/32 and IOP at 26 mmHg under treatment. The slit lamp examination after dilatation revealed the absence of dexamethasone implant in the visual axis (Fig. 2).

## Discussion

Although complications of dexamethasone implant injections are rare, cases of retinal and vitreous hemorrhages after traumatic impact of a dexamethasone implant in vitrectomized eyes, or migration of the implant in the anterior chamber of the eye are described in the literature. To the best of our knowledge, this case of an implant confined in Berger’s space was not previously described. Berger’s space accumulation of pigment was already reported in cases of pigmentary glaucoma (Nagarajaiah and Shun-Shin 2011). It was observed that in case of break in Wieger’s ligament, corresponding to the connexion between the edges of the anterior vitreous limiting membrane and the periphery of the posterior surface of the lens, that pigment could flow in Berger’s space and accumulate there. Berger’s space corresponds to the space between the anterior hyaloid and posterior lens capsule. We suggested that for our patient, the implant lodged in this space after break of the Wieger’s ligament and stayed confined in this localization. Cataract surgery probably fragilized the Wieger’s ligament still intact and allowed the implant to move to a less inconvenient location for our patient.

## Conclusions

We describe here the confinement of dexamethasone implant in Berger’s space in the visual axis, a yet undescribed complication after intravitreal injection.
